# Effect of nonsurgical periodontal therapy on the level of salivary antioxidants in patients with generalized moderate-to-severe chronic periodontitis

**DOI:** 10.15171/japid.2019.004

**Published:** 2019-08-31

**Authors:** Ferena Sayar, Roya Shariatmadar Ahmadi, Mostafa Montazeri

**Affiliations:** ^1^Department of Periodontics, Faculty of Dentistry, Islamic Azad University Tehran Branch, Iran; ^2^Periodontist, Private Practice, Shiraz, Iran

**Keywords:** Antioxidants and periodontitis, dental scaling, oxidants, periodontitis, root planing

## Abstract

**Background:**

In the course of periodontal diseases, polymorphonuclear leukocytes (PMNs) produce oxidative agents and free radicals, thus triggering oxidant-antioxidant disequilibrium in the saliva. Due to the reduction of antioxidant protective effect, oxidative stress is induced, destroying periodontal tissues. This study aimed to investigate the consequences of the non-surgical phase of periodontal therapy on the level ofsalivary antioxidantsin patients with generalized moderate-to-severe chronic periodontitis.

**Methods:**

Un-stimulated salivary samples were collected from 43 patients with generalized moderate-to-severe chronic periodontitis for 5 minutes. Clinical parameters, including clinical attachment loss (CAL), bleeding on probing (BoP) and pocket depth (PD), were recorded in each tooth and subsequently, scaling and root planing (SRP) was carried out. After four weeks, salivary samples were collected once again, and the above-mentioned clinical parameters were recorded. Following centrifugation and freezing at a temperature of -80°C, salivary samples were examined simultaneously in a single day, and the level of their antioxidants was measured with ferric reducing ability of plasma (FRAP) method using a spectrophotometer.

**Results:**

The concentration of salivary antioxidants significantly increased four weeks following the non-surgical periodontal therapy (P<0.0001). Moreover, the clinical parameters of CAL, BoP and PD showed a significant decrease in 4 weeks as well (P<0.0001).

**Conclusion:**

The level of salivary antioxidants in patients with generalized moderate to severe chronic periodontitis significantly increased after etiotropic periodontal therapy, indicating the possible beneficial influence of periodontal therapy on the level of salivary antioxidants in patients suffering from periodontitis.

## Introduction

Periodontitis is an inflammatory condition which occurs as a result of the complex interaction between periodontal pathogenic bacteria and host immune system, leading to the destruction of periodontal attachment and alveolar bone with the subsequent pocket formation and gingival recession or both.^1,2^ The excessive response of the host to the bacteria results in the secretion of matrix metalloproteinases, osteoclastogenic factors and proinflammatory cytokines, which are responsible for the majority of tissue destruction.^3-5^ One of the consequences of tissue damage is an increase in the number and activity of polymorphonuclear cells (PMNs) residing in gingival crevicular fluid (GCF) and the subsequent release of their products.^5^ Oxygen free radicals (reactive oxygen species; ROS) such as superoxide anion (O_2_-), hydrogen peroxide (H_2_O_2_) and nitric oxide (NO) are some of these noxious substances produced by different cells. These include PMNs (in the process of phagocytosis of bacteria), fibroblasts and osteoclasts, destroying both the host cells and microorganisms.^6-8^ ROS are molecular mediators with one or more unpaired electrons that cause damage to DNA and proteins, lipid peroxidation, oxidation of enzymes and stimulation of monocytes and macrophages to secrete more proinflammatory cytokines. Thus, the oxidant‒antioxidant imbalance may occur due to lipid peroxidation, causing damage to the cellular integrity and eventually destroying the connective tissue and bone surrounding the teeth.^9-11^

Antioxidants are the primary mechanisms protecting the body against oxidants (ROS) and oxidative stress and are released by PMNs (such as neutrophils) in inflammation sites and could be detected in the secretions of submandibular and parotid glands under standard conditions (i.e. uric acid and peroxidase).^12-14^ Periodontal pathogens such as *Porphyromonas gingivalis* exhibit the ability to resist the bactericidal activity of neutrophils' free radicals by secreting their peculiar antioxidants.^15,16^ There is a state of equilibrium between oxidants and antioxidants in the saliva of periodontally healthy individuals; in the case of disease, this balance progresses towards oxidants.^10,13^

Measuring the total antioxidant capacity of the saliva is a routine method in various studies.^6,9,15,17-23^ In a research by Chapple et al,^18^ superoxide dismutase (SOD) decreased in deep periodontal pockets, but following the first phase of periodontal therapy, the level of total antioxidants in the gingival crevicular fluid (GCF) increased. Similar to this study, Diab-Ladki et al,^9^ Sculley et al^15^ and Brock et al^19^ reported that the total antioxidant capacity of the saliva of periodontally compromised patients decreased. In contrast, Kim et al^6^ reported that the GCF antioxidants' levels showed no significant difference before and after the first phase of periodontal therapy. Liskmann et al^20^ also reported a decrease in the levels of antioxidants around the implants suffering from peri-implantitis.

This study aimed to assess the impact of the etiotropic phase of periodontal therapy (scaling and root planing; SRP) on the level of salivary antioxidants in patients with generalized advanced chronic periodontitis.

## Methods

### Ethical Approval

This research on human samples complied with all the relevant national regulations and with those approved by "Faculty of Dentistry, Islamic Azad University of Tehran" and was registered at the Iranian Clinical Trials (IRCT) website under the code IRCT2017052717053N6.

### Study Population

This study randomized clinical trial (RCT) was conducted on 43 patients, including 24 females and 19 males aged 28-67 years old, visiting the Department of Periodontics, Tehran Dental Branch of Islamic Azad University from May 2014 to April 2015. The subjects were selected as target-based consecutive samples and a consent form was obtained from each participant after explaining the research protocol to them. All the subjects were systemically healthy with at least 15 teeth present and did not have a history of taking any antibiotics, immunosuppressive drugs, NSAIDs, vitamins, food supplements, tobacco and narcotics in the last three months before the study. Moreover, pregnant and lactating women were excluded from the study due to the possibility of GCF and saliva alterations.

### Periodontal Examination

Generalized moderate-to-severe chronic periodontitis was diagnosed in the subjects according to the following criteria: clinical attachment loss (CAL)≥3 mm in more than 30% of the examined sites around each tooth. Six locations were tested around each tooth: mesiobuccal, mid-buccal, distobuccal, mesiolingual, mid-lingual and distolingual for radiographic bone loss >30% of the root length. Bleeding on probing (BoP) was determined by Bay and Ainamo index.^24^ Pocket depth was also measured using a Williams probe as the distance between the gingival margin and the bottom of the pocket at six sites above.

Ten patients were examined twice by a single periodontist with a 1-hour interval to determine intra-examiner reproducibility for the variables (CAL, PD and BOP). The intraclass correlation coefficient was found to be 90%.

### Saliva Sampling

Before starting the non-surgical phase of periodontal therapy, un-stimulated saliva was collected in test tubes from all the subjects for 5 minutes between 10 and 12 a.m. The samples were kept in dry ice, centrifuged at 4°C for 10 minutes at 4000 rpm and stored at -80°C.

### Non-surgicalPeriodontal Therapy

Following saliva sampling, the clinical parameters, including pocket depth (PD), clinical attachment loss (CAL) and bleeding on probing (BoP) were recorded and the patients underwent the non-surgical phase of periodontal therapy. Throughout a session, scaling and root planing (SRP) by hand scalers and the ultrasonic device was carried out, and the patients were instructed in proper brushing (according to Bass method) and flossing. After four weeks (one month), clinical parameters were re-recorded, and saliva was extracted from the patients according to the stated plan.

### Laboratory Tests on Saliva

The levels of salivary antioxidants in all the samples of both periods were measured in a single day with the FRAP (Ferric Reducing Ability of Plasma) method by Pharmacia Biotech Ultrospec 3000 UV spectrophotometer. Benzie et al^25^ method determined the antioxidants by reducing ability, which was used to measure the total antioxidant capacity of plasma. In this approach, Fe^3+^-TPTZ complex (2,4,6-Tri(2-pyridyl)-s-triazine, Sigma-Aldrich Co.) is reduced to Fe^2+^-TPTZ which produces a blue color in acidic pH and has the maximum wavelength absorption in 593 nm. In the presence of a reducing compound in the saliva, the blue color will be established. The higher the reducing power the test sample has, the faster will be the reaction rate and color production.^25^

Acetate buffer is required for measuring the total antioxidant capacity of the saliva. 3.1 g of sodium acetate was mixed with 16 mL of acetic acid and increased to a volume of 1 liter with distilled water; then the pH was adjusted to 3.6 to prepare the TPTZ solution. Also, 10 mmol of TPTZ was dissolved in 40 mmol of hydrochloric acid. The primary reactant solution was prepared by mixing 2.5 mL of TPTZ, 2.5 mL of iron chloride and 25 mL of acetate buffer.

During the test, the temperature of the prepared solution was raised to 37°C, and then 900 microliters of the solution were poured in the cuvette, and the spectrophotometer was calibrated at a wavelength of 593 nm. Then, the reaction was started by adding 30 microliters of saliva previously centrifuged at 4°C. The absorption was read at the beginning of the reaction and after 8 minutes. The difference between the initial and final absorptions (ΔA=593 nm) was calculated for each sample, and its value was determined using the standard curve.

To graph the standard curve, we used iron sulfate. Several iron sulfate solutions with a concentration of 100‒1000 μmol/L were prepared, and their absorption was determined separately using the reactant solution. Using the obtained values, we used the regression curve, and with the regression equation, the concentration of each sample was determined.

Finally, the test result was reported in μmol/L, which is in fact the amount of yielded Fe^2+^. As a control, the test was conducted with saliva, but without adding Fe^3+^, and no color changes were observed. This suggests that saliva containing EDTA does not have Fe^2+^ or other factors that directly react with TPTZ. In the second control test, all the reaction components were present, but the distilled water was added instead of saliva. No absorption was observed in this process, which indicates that in the absence of saliva, the reagent solution lacks any factors that would cause conversion of Fe^3+^ to Fe^2+^.

### Statistical Analysis

Data obtained from the regression equation was the basis for the statistical analysis. To test the study hypotheses, 95% confidence intervals were determined for the mean population value, and Smirnov-Kolmogorov test and paired t-test were used. Judgment criteria for each study hypothesis in each test were the P-value and its comparison with α=0.05. These calculations were performed with SPSS 16.

## Results

This randomized clinical trial (RCT) was performed on 43 patients (24 females and 19 males) aged 28-67 years old (with a mean age of 48.30±10.90 years) suffering from generalized moderate-to-severe chronic periodontitis.

Statistical indices for CAL, BoP and PD before and after the therapy are presented in Table 1.

**Table 1:** Statistical indices for CAL, BoP and PD, before and after the therapy

**Indices**	**Number**	**Mean**		**Standard deviation**		**95% Confidence interval** **Minimum Maximum**				**P-value**
**Variables**		**Before**	**After**	**Before**	**After**	**Before**	**After**	**Before**	**After**	
**CAL**	43	3.75	3.16	0.709	0.616	3.05	2.24	6.66	5.53	0.0001
**BoP**	43	46.18	46.18	11.90	5.02	25	7	70	30	0.0001
**PD**	43	2.72	2.72	0.478	0.439	1.78	1.51	4.22	3.80	0.0001

According to the above table, significant differences were observed between the numeric values of CAL, BoP and PD parameters in the patients before and after the therapy. All the parameters decreased significantly following the non-surgical phase of periodontal treatment (P<0.0001).

Table 2 presents the total antioxidant concentrations before therapy and one month afterwards.

**Table 2:** Statistical indices of antioxidant concentrations before and after the therapy

**Groups**	**Mean**	**Standard deviation**	**95% Confidence interval** **Minimum Maximum**		**P-value**
**Before**	184.22	63.27	96.43	336.43	0.0001
**One month after the therapy**	332.94	75.66	169.29	509	

Based on the above table, the number of antioxidants one month following the etiotropic phase of periodontal therapy increased significantly (P<0.0001).

**Figure 1:**
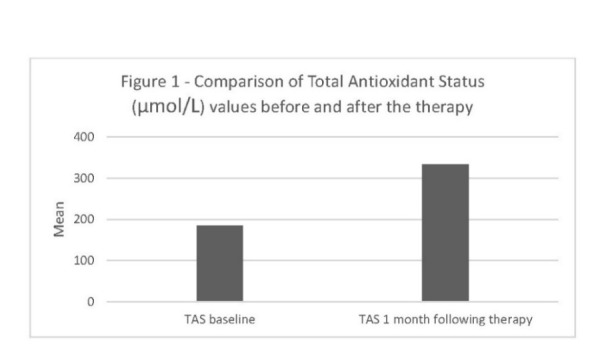
Comparison of total antioxidant concentrations in the saliva (µmol/L) before and after therapy.

**Figure 2:**
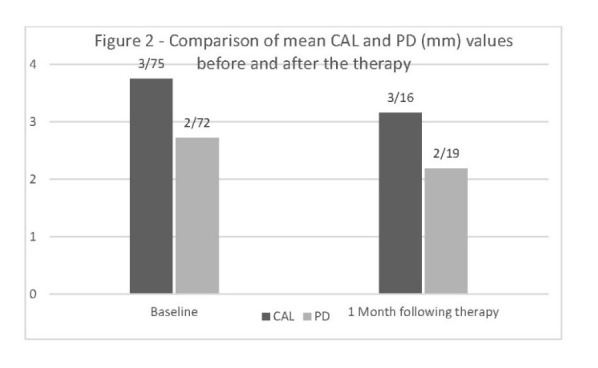
Comparison of mean CAL and PD (mm) values before and after therapy.

**Figure 3:**
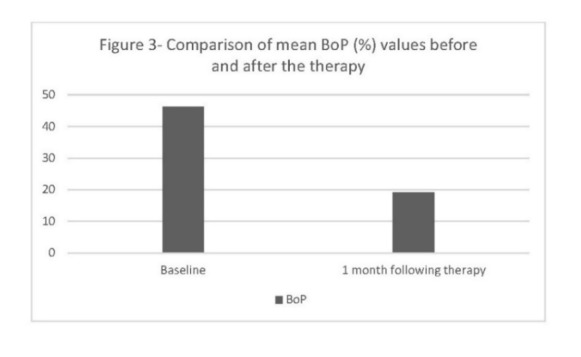
Comparison of mean BoP (%) values before and after therapy.

Figures 1, 2 and 3 demonstrate the concentrations of antioxidants and CAL, BoP and PD parameters before and after the treatment.

## Discussion

In this randomized clinical trial on 43 patients with generalized advanced chronic periodontitis, a statistically significant difference was observed between the concentrations of salivary antioxidants, before and following the etiotropic phase of the periodontal therapy, with significant increases in antioxidant levels after treatment. This finding reflects the improvement in antioxidant status after scaling and root planing and is in fact an indicator of a reduction in oral microbial load. In the process of periodontal disease, oxidant‒antioxidant equilibrium progressed towards the increase in oxidants, thereby causing tissue destruction.^10,13^

Neutrophils exposed to periodontal pathogens produce oxygen free radicals that, in addition to killing microorganisms, can also cause tissue injury and this destructive effect is neutralized by the body's antioxidant system.^14^ If under any circumstances such as microbial invasion, smoking or malnutrition, the reducing ability of the antioxidant system is decreased, the oxidant‒antioxidant equilibrium will be disturbed.^1^ As a result, the antioxidant system will not be able to eliminate all the ROS molecules, and the disease will take a destructive course.^13^ It is assessed that between 1 and 3 billion ROS molecules are produced daily in the body, which is controlled by the antioxidant system.^18^

In almost all the studies on the antioxidants, the total antioxidant capacity has been assessed.^6,9,17,19,26^ The reason might be the fact that the study of the total antioxidant capacity is more comfortable and cheaper and takes less time. Meanwhile, the study of individual antioxidants is not possible in some cases, and it is not cost-effective. Moreover, the study of every single antioxidant regardless of their total concentrations yields results that are far from reality and sometimes false.

In various studies^9,12,15,19,22,27,28^ on total salivary antioxidant levels in patients with periodontitis, the antioxidant levels were lower compared to healthy individuals, consistent with the findings of this study. A recent survey by Villa-Correa et al^29^ showed a significant correlation between salivary levels of glutathione reductase and periodontal clinical parameters, indicating the importance of oxidative stress processes in periodontal diseases.

Liskmann et al^20^ showed that the total salivary antioxidant levels around implants with mucositis or peri-implantitis were significantly lower than healthy implants. However, Surdacka et al^30^ studied the relationship between clinical parameters and antioxidant status in diabetic pregnant women and observed that in addition to unfavorable gingival and periodontal indices in diabetic women compared to healthy women, the total amount of salivary antioxidants also increased in diabetic women. The authors attributed this observation to the increase in oxidative stress as a result of catalase activity in diabetic patients. The differential concentration of salivary antioxidants such as uric acid (known as the primary antioxidant in saliva), had not changed in that study. One reason for the inconsistency of these results with other studies might be the fact that in this study, pregnant women who were diagnosed with gestational diabetes and women who had diabetes up to 19 years before the pregnancy were mixed.

Shirzaiy et al^26^ showed that following the initial phase of periodontal therapy, the total antioxidant capacity of the saliva significantly increased. It was also reported that although insignificant, the increase in total antioxidant capacity was more pronounced in men and specific age groups.

In contrast, a research by Moore et al^31^ on the antioxidant activity of saliva and periodontal tissue showed no difference in antioxidant levels and activity between healthy subjects and periodontitis patients. The authors concluded that this was due to the increase in GCF as a result of gingival inflammation and because of increased levels of antioxidants such as peroxidase. Also, the lactoferrin exerts protective effects on oral tissues against free radicals. It is of great value to mention that the patients studied were only seven subjects and the most important antioxidant in saliva was identified to be uric acid.

Consistent with the results of this research, Muniz et al^32^ reported that non-surgical periodontal treatment significantly reduced oxidative stress markers such as melanodialdehyde (MDA). Thus, it was concluded that periodontal intervention could be beneficial for controlling systemic oxidative stress. In fact, by blocking the arachidonic acid cascade, new treatment strategies can be introduced for controlling the inflammation caused by oxidative stress.

In a study by Chapple et al^18^ on the antioxidant activity in patients with periodontitis, total serum antioxidant levels in healthy subjects and patients with severe or mild periodontitis were not different, but in GCF, the antioxidants levels in periodontal patients were less than healthy subjects. Following scaling and root planing, the total antioxidant levels increased and reached the control group levels. Moreover, serum total antioxidant levels were higher in men than in women, but its levels in GCF were not different between the two genders. The difference in total antioxidant levels in the GCF, before and after therapy, was similar to the changes in the present study. However, in Chapple’s study, the number of studied groups was small. Nontheless, the results were statistically significant before and after the therapy.

In a study by Sculley et al,^15^ the total salivary antioxidant capacity, regardless of periodontal status, was lower in women than in men, which was attributed to the lower levels of ascorbic acid and uric acid in women than in men. In addition, antioxidant levels in subjects with periodontal disease, especially urea, were lower than that of healthy subjects.

A study by Ongoz et al^33^ showed that one month following initial periodontal therapy, levels of 8-hydroxy-deoxyguanosine (8-OHdG) and periodontal clinical parameters significantly decreased in both normal-weight and obese patients. This finding suggests that initial periodontal treatment has positive effects on antioxidant levels regardless of patient weight. In addition, a recent study by Ling et al^34^ showed that following initial periodontal therapy, neutrophilic superoxidase significantly decreased, consistent with the current research, indicating that free radicals are diminished after periodontal treatment.

In a study by Mousavi Jazi et al,^35^ researchers observed a significant correlation between pocket depth and total antioxidant capacity of GCF and melanodialdehyde (MDA). However, there was no difference between peri-implantitis-affected and healthy areas regarding total antioxidant capacity, MDA and superoxidase dismutase (SOD). The authors concluded that measuring the oxidative stress markers is not useful in differentiating the peri-implantitis with healthy implant conditions. The results of this study probably reflect the fundamental differences in peri-implant mucosa and gingival sulcus around teeth, necessitating further research.

Given the significant changes in total salivary antioxidant levels after periodontal therapy, it can be concluded that ROS production has a prominent role in the development and progression of periodontal disease and controlling them can be facilitated through timely diagnosis and treatment of periodontal disease.

## Conclusion

Within the limitations of this study, it was observed that salivary antioxidant levels significantly increased after an etiotropic phase of periodontal therapy, indicating a positive effect of treatment on the salivary antioxidant levels in patients with generalized advanced chronic periodontitis.

### Implications

One of the constraints of this study was the lack of pre-prepared kits needed to measure the antioxidant levels in Iran, which prompted us to change the methodology and spend more time. Moreover, conducting a study with larger sample size and advanced periodontal therapy is recommended to make the results more decisive.

### Suggestions

The following topics are suggested for future research:

1. Studying the distinctive levels of antioxidants in patients with periodontitis before and after an etiotropic phase of the therapy.

2. Comparison of salivary antioxidant levels in various periodontal diseases such as gingivitis and periodontitis.

3. Studying the effects of periodontal surgery on the salivary antioxidant levels.

## Conflict of Interests

The authors explicitly declare no conflict of interests in connection with this article.
